# Correction to ‘Magnetic signals from oceanic tides: new satellite observations and applications’

**DOI:** 10.1098/rsta.2025.0045

**Published:** 2025-05-08

**Authors:** Alexander Grayver, Christopher C. Finlay, Nils Olsen

**Affiliations:** ^1^ Institute of Geophysics and Meteorology, University of Cologne, Cologne, Germany; ^2^ DTU Space, Technical University of Denmark, Lyngby, Hovedstaden, Denmark

**Keywords:** correction

The phases of the KALMAG and CM6 models were not rendered relative to the Greenwich meridian, resulting in erroneous values in table 2 and figure 5. We have now corrected the RMSE (table 2) and correlation coefficient (figure 5) values using the consistent phases for these two models.

**Figure 5.**
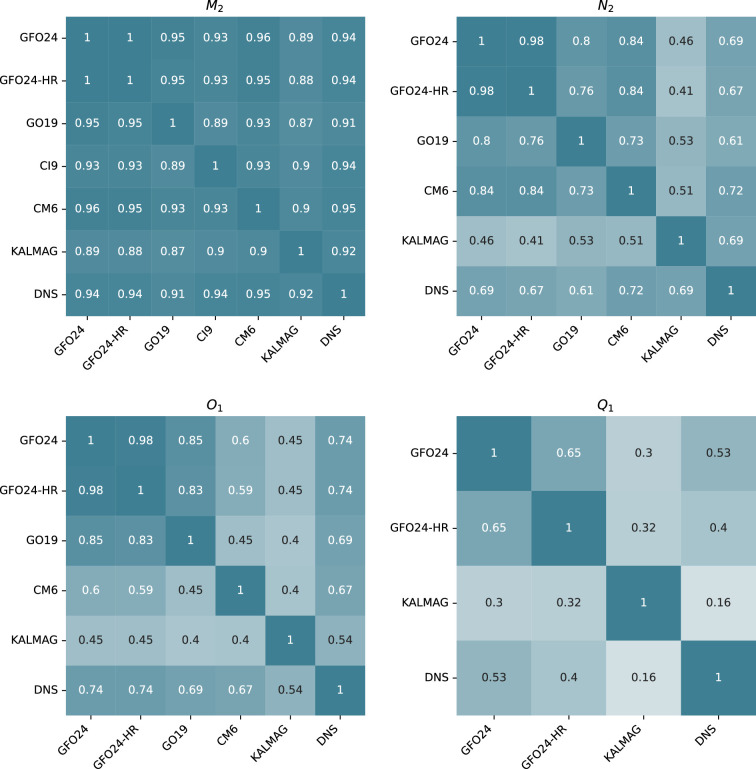
Pearson correlation coefficients between different models of the observed signals and a three-dimensional DNS for the four tidal constituents. Correlations were calculated between real parts of the radial magnetic field maps at the altitude of 430 km (shown in figures 2 and 3). Correlation coefficients for imaginary parts are similar.

**Table 2.** (a) RMSE between observed tidal signals and three-dimensional DNS for four considered tidal constituents (calculated as described in §2b). The radial magnetic field component at an altitude of 430 km was used. (b) Corresponding maximum SH degree used to calculate the magnetic fields.

(a) RMSE (nT)	$M_{2}$	$N_{2}$	$O_{1}$	$Q_{1}$
GFO24	0.166	0.063	0.070	0.027
GFO24-HR^a^	0.167	0.069	0.074	0.033
GO19	0.193	0.088	0.098	—
CI9	0.169	—	—	—
CM6^b^	0.196	0.089	0.126	—
KALMAG	0.210	0.088	0.092	0.062

(b) *N*_max_	$M_{2}$	$N_{2}$	$O_{1}$	$Q_{1}$
GFO24	28	12	12	4
GFO24-HR^a^	32	14	14	6
GO19	28	12	12	—
CI19	18	—	—	—
CM6^b^	28	12	12	—
KALMAG	30	39	30	30

^a^ Higher-resolution models, determined up to higher SH degrees compared to GFO24.

^b^ Although higher-degree expansions were reported in the original study [34], the authors stated that only signals up to the herein specified degrees are reliable.

These revisions do not affect the results, conclusions or overall purpose of this paper.

The table and the figure have been corrected in the version of record.

